# IGF2/H19 hypomethylation in a patient with very low birthweight, preocious pubarche and insulin resistance

**DOI:** 10.1186/1471-2350-13-42

**Published:** 2012-05-30

**Authors:** Rinki Murphy, Lourdes Ibáñez, Andrew Hattersley, Jörg Tost

**Affiliations:** 1 Faculty of Medical and Health Sciences, University of Auckland, Auckland, Private Bag 92019, New Zealand; 2 Endocrinology Unit, Hospital Sant Joan de Déu, University of Barcelona, Barcelona, and Centro de Investigación Biomédica en Red de Diabetes y Enfermedades Metabólicas Asociadas (CIBERDEM), ISCIII, Madrid, Spain; 3 Institute of Biomedical and Clinical Sciences, Peninsula Medical School, Exeter, UK; 4 Laboratory for Epigenetics, Centre National de Génotypage, CEA-Institut de Génomique, Evry, France

**Keywords:** Insulin like growth factor 2, Intrauterine growth restriction, Short stature, Insulin resistance

## Abstract

**Background:**

Insulin like growth factor 2 (*IGF2*) is an imprinted gene, which has an important role in fetal growth as established in mice models. IGF2 is downregulated through hypomethylation of a differentially methylated region (DMR) in Silver Russell syndrome (SRS), characterised by growth restriction. We have previously reported that severe pre- and post-natal growth restriction associated with insulin resistance and precocious pubarche in a woman without body asymmetry or other SRS features resulted from a balanced translocation affecting the regulation of her *IGF2* gene expression. We hypothesised that severe pre- and post-natal growth restriction associated with insulin resistance and precocious pubarche in the absence of SRS are also caused by downregulation of *IGF2* through hypomethylation, gene mutation or structural chromosomal abnormalities.

**Methods:**

We performed routine karyotyping, IGF2 gene sequencing and investigated DNA methylation of the *IGF2* differentially methylated region (DMR)0 and *H19* DMR using pyrosequencing, in four women selected for very low birth weight (<−3 SDS for gestational age), precocious pubarche, short adult stature (<−2 SDS), and insulin resistance (defined as HOMA-IS < 80%); and compared their methylation results to those of 95 control subjects.

**Results:**

We identified a 20 year old woman with severe hypomethylation at both DMRs. She was the smallest at birth (birthweight SDS,-3.9), and had the shortest adult height (143 cm). The patient was diagnosed with polycystic ovarian syndrome at the age of 15 years, and had impaired fasting glucose in the presence of a low BMI (19.2 kg/m^2^).

**Conclusions:**

Our case of growth restriction, premature pubarche and insulin resistance in the absence of body asymmetry or other features of SRS adds to the expanding phenotype of *IGF2/H19* methylation abnormalities. Further studies are needed to confirm whether growth restriction in association with premature pubarche and insulin resistance is a specific manifestation of reduced *IGF2* expression.

## Background

**Figure 1:**
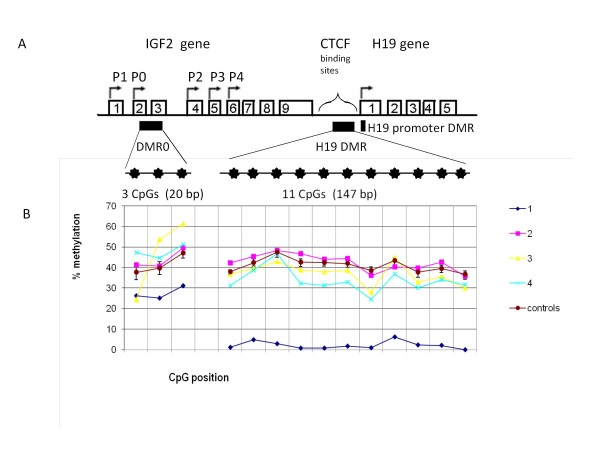
**A Structural characteristics of the human*IGF2*/* H19 *locus with nine exons and five exons for* IGF2 *and* H19 *genes respectively.** Transcription start site and promoters used are indicated by arrows. Regions of differentially methylated regions (DMRs) are shown with black bars. For each DMR, the number of CpGs analysed is indicated and their relative positions are represented by filled stars corresponding to the methylation site horizontal axis of Figure 1**B**. **B** Graph of methylation patterns of the *IGF2*/*H19* locus (expressed as percentage for each CpG) for each case and mean with 95% confidence interval for the 95 control samples.

Aberrant imprinting of the *IGF2*/*H19* locus on chromosome 11p15.5 has a crucial role in Beckwith-Wiedemann syndrome (BWS, OMIM #130650) [1], Silver-Russell syndrome (SRS, OMIM#180860) [2,3] and several human cancers [4,5]. Paternal *IGF2* and maternal *H19* expression in humans, correlates with the close regulation in mice, by two differentially methylated regions (DMRs). Both are normally methylated on the paternally inherited allele: *IGF2* DMR0 (located between exons 2 and 3 of *IGF2*), and *H19* DMR, located 4 kb upstream of the transcription start site of *H19*[6], resulting in around 40-50% methylation at each of these sites [7-9] (Figure 1 A). The *H19* DMR locus is also known as the imprinting control region (ICR) of the *IGF2H19* locus because it is the centre from where methylation on the paternal allele is propagated, including the IGF2 DMR0, H19 promoter DMR (which directly silences H19 expression from the paternal allele), in addition to containing the recognition sites for CCCTC binding factor (CTCF). CTCF is only able to bind to the unmethylated H19 DMR on the maternal allele which blocks the access of maternal allele IGF2 promoters from its downstream enhancers, but enables H19 expression from this allele [10].

Hypermethylation of the *H19* DMR on the maternal allele associated with *IGF2* over-expression is found in 10% of patients with neonatal overgrowth and transient neonatal hypoglycemia characteristic of BWS [1], while hypomethylation of the *H19* DMR on the paternal allele (associated with *IGF2* under-expression) is found in over 50% of patients with SRS [9,11], characterized by intrauterine and post-natal growth restriction. In a study of 70 BWS and 2 SRS patients, the methylation patterns at the *IGF2* DMR0 resembled that at the *H19* DMR, in contrast to 33 non-syndromic Wilms tumours in which *IGF2* DMR0 methylation was negatively correlated with *H19* DMR methylation [12]. Selective hypomethylation for either the *H19* DMR or the *IGF2* DMR0 has been reported in 13 patients with SRS [13]. The etiological role of *IGF2* in regulating intrauterine growth and tumour formation has been established in animal studies [14,15]; however its role in the other features of BWS (such as exomphalos, macroglossia, ear abnormalities, hemihyperplasia) and SRS (craniofacial abnormalities, clinodactyly, genital abnormalities) is unknown, but might relate to the wide range of tissues in which *IGF2* is expressed.

Besides imprinting abnormalities, structural chromosomal abnormalities affecting the *IGF2/H19* locus have been described: Maternal 11p15 duplications (predicted to decrease IGF2 expression through increased doseage of maternal suppressor sequences) have been found in growth restricted patients [16,17], while paternal 11p15 duplications (predicted to increase IGF2 expression through relatively reduced doseage of maternal suppressor sequences) have been associated with BWS patients [1]. We previously reported a woman with severe intrauterine growth restriction (birthweight SDS −5.4), short stature and a balanced chromosomal translocation breakpoint t(1,11)(p36.22;p15.5), disrupting the access of her paternally inherited *IGF2* allele from its telomeric enhancer sequences [18]. Although she was predicted to have paternal *IGF2* under-expression similar to those with SRS, she did not fit the diagnostic criteria for SRS, beyond the obligatory pre- and post-natal growth restriction, including lack of any dysmorphisms supported by normal childhood photographs [18]. In addition, she had insulin resistance and precocious pubarche (pubic hair before the age of 8 yrs), two characteristics not commonly reported in association with SRS [18]. Due to the phenotype of this case, we sought to identify additional cases of paternal *IGF2* inactivation through either structural chromosomal abnormalities, *IGF2* gene mutations or *IGF2/H19* methylation abberrations, by screening women with similar characteristics of intrauterine growth restriction, short stature, insulin resistance and precocious pubarche. Here we provide this data including detailed methylation patterns of both *IGF2* DMR0 and *H19* DMR in 4 women and in 95 control subjects from a healthy population.

## Methods

### Participants

The study population consisted of four girls (age, 13–20 yr) recruited from a tertiary Spanish hospital without any congenital abnormalities or dysmorphism, selected on the following criteria:

· Birthweight standard deviation score (SDS) below −3.0 after a term gestation not complicated by gestational diabetes, preeclampsia or use of alcohol or drugs;

· A history of precocious pubarche attributable to premature adrenarche, as judged by increased dehydroepiandrosterone-sulfate (DHEAS) and/or androstenedione levels for chronological age [19].

· Adult height SDS below −2.0 for Spanish population standards, with closed epiphysis.

· Decreased fasting insulin sensitivity, estimated from fasting insulin and glucose levels using the homeostasis model assessment (HOMA-CIGMA Calculator program, version 2.00), and defined as a HOMA-IS value less than 80% [20].

None of the patients had thyroid dysfunction or late-onset congenital adrenal hyperplasia; none were receiving any medication known to affect gonadal function or carbohydrate metabolism. At the time of the study, all were at least 2 yr beyond menarche (mean, 6.5 years).

In all cases, we undertook karyotyping analysis, screened for mutations in the *IGF2* gene and performed methylation analysis of the *H19* DMR, and *IGF2* DMR0. The study also involved 95 control patients enrolled in the Exeter Family Study of Childhood Health (EFSOCH) [21] for comparative methylation analysis of the *IGF2*/*H19* locus.

### Ethical approval

The study was approved by the Institutional Review Boards of the participating hospitals. Informed consent was obtained from parents and/or the subjects.

### Assessments

Birth weight, birth length, gestational age and current height were obtained from hospital records, and SDS were derived from local reference values [22].

Serum glucose was measured by the glucose oxidase method. Immunoreactive insulin was assayed by IMX (Abbott Diagnostics), and serum sex hormone-binding globulin (SHBG), testosterone, androstenedione and DHEAS were assayed by immunochemiluminiscence (IMMULITE 2000, Diagnostic Products, Los Angeles, CA), as described [23]; the intra- and inter-assay coefficients of variation (CVs) were between 4% and 8%. The free androgen index, equivalent to free testosterone, was calculated as total testosterone/(nmol/L)/SHBG (nmol/L) x 100. The samples were kept frozen at −20°C until assay.

### DNA sequencing

Leucocyte derived DNA from all study participants was sequenced for *IGF2* mutations as previously described [18].

### DNA methylation analysis

500 ng Genomic DNA (derived from peripheral blood leucocytes) was treated with sodium bisulphite and then 50 ng of this DNA was used as a template for PCR amplification. The three DMRs were analyzed: *IGF2* DMR0 (3 CpGs), and in the 3^rd^ CTCF binding site of the *H19* DMR (11 CpGs) by pyrosequencing as previously described [24]. Accession numbers and nucleotide positions of each DMR, PCR primers and annealing temperatures as well as the size of PCR products have been detailed previously [25].

### Statistical analysis

Methylation levels at *IGF2* DMR0 (3 CpGs), and *H19* DMR (11CpGs) in the four cases are graphed with corresponding median and IQR methylation levels of the 95 control EFSOCH samples (Figure 1B).

## Results

SRS was not diagnosed in any of these women, based on absence of typical facial features, body asymmetry, clinodactyly or brachydactyly, genital abnormalities, café au lait spots, feeding difficulties or delayed speech. However, two women (subjects 1 and 3) had less specific features of down-turned corners of their mouth, and subject 1 also had retrognathia and a squeaky voice (Table 1). All women experienced early menarche (range, 10–11 yr) as compared with population standards [26]. Subjects 1 and 3 were diagnosed with polycystic ovary syndrome after menarche, defined as the presence of hirsutism [Ferriman & Gallway score > 8], oligomenorrhea (menstrual cycles > 45 days), and increased testosterone, androstenedione and/or free androgen index (9). Insulin sensitivity (HOMA-IS %) ranged from 18% to 65% and was thus well below normal limits in all subjects (reference mean ± SEM from age-matched healthy volunteers [20]: n = 24; age, 15.3 ± 0.2 yr; 104% ± 5).

**Table 1:** Clinical characteristics of women with low birthweight, precocious pubarche, short stature and insulin resistance

**Subject**	**1**	**2**	**3**	**4**
Age (yr)	20	15	20	13
Gestational age (wk)	39	38	40	37
Birthweight SDS (Kg)	−3.9 (1.8)	−3.1 (1.8)	−3.0 (2.2)	−3.2 (1.2)
Birth length SDS (cm)	−3.9 (42)	−3.1 (42)	−3.0 (45)	−3.2 (39)
Head circumference SDS (cm)	−1.8 (31.5)	−1.8 (30.5)	−2.5 (31.0)	n/a
Mid-parental target height Z-score (cm)	−0.4 (161)	−0.8 (159)	−1.5 (155)	0.2 (164)
Adult height Z-score (cm)	−3.6 (143)	−2.1 (153)	−2.3 (151)	−2.3 (151)
Adult weight (Kg)	39.3	56.0	68.0	41.7
Body mass index SDS (Kg/m^2^)	−0.7 (19.2)	0.8 (23.9)	2.6 (29.8)	−1.0 (18.3)
Age at pubarche (yr)	7.0	7.8	6.0	7.5
Age at menarche (yr)	11.0	10.0	11.0	10.0
Polycystic ovary syndrome (age, yr)	15.0	No	15.0	No
Body asymmetry	No	No	No	No
Triangular shaped face	No	No	No	No
Irregular crowded teeth	No	No	No	No
Low set ears	No	No	No	No
Retrognathia	Yes	No	No	No
Prominent forehead	No	No	Yes	No
Down-turned corners of the mouth	Yes	No	Yes	No
Thin lips	No	No	No	No
Squeaky voice	Yes	No	No	No
Genital abnormalities	No	No	No	No
Clinodactyly or brachydactyly	No	No	No	No
Glucose (mg/dl)	102 ↑	88	81	83
Insulin (mU/L) ^†^	20.4 ↑	17.9 ↑	16.6 ↑	17.4 ↑
Insulin sensitivity (% HOMA) ^†^	65 ↓	58 ↓	54 ↓	57 ↓
Testosterone (ng/dL) ^†^	65 ↑	64 ↑	84 ↑	60 ↑
SHBG (μg/dL) ^†^	29 ↑	31 ↑	15 ↑	27 ↑
Free Androgen Index ^†^	7.8 ↑	7.2 ↑	19.4 ↑	7.7 ↑
Androstenedione (ng/dL) ^†^	337 ↑	190	567 ↑	221 ↑
DHEAS (μg/dL) ^†^	329 ↑	86	311 ↑	89
Karyotype	Normal	Normal	Normal	Normal
*IGF2* gene mutation	No	No	No	No
H19 DMR % (ref range 33.0 – 49.8)^#^	2.2 ↓	42.3	36.9	33.7
*IGF2* DMR0% (ref range 36.5 – 54.9)^#^	27.6 ↓	43.9	46.4	47.7

SHBG, sex hormone-binding globulin; DHEAS, dehydroepiandrosterone-sulfate; free androgen index, testosterone (nmol/L) x SHBG (nmol(L)/100; n/a, not available.

^†^ Reference data (mean ± SEM) from age-matched healthy volunteers[20]: n = 24; age, 15.3 ± 0.2 yr; glucose: 79 ± 1.8 mg/dL; insulin: 11.3 ± 1.0 mU/L; insulin sensitivity (% HOMA): 104 ± 5; testosterone: 31 ± 3 ng/dL; SHBG: 1.9 ± 0.1 μg/dL; free androgen index: <5; androstenedione: 156 ± 14 ng/dL; DHEAS: 125 ± 12 μg/dL.

# Reference range for % methylation, stated as 95% confidence interval derived from 95 control patients.

↑ and ↓ are used in the table to indicate clearly increased and decreased laboratory values.

Subject 1 with the lowest birth weight SDS (−3.9) and shortest adult height of 143 cm (−3.6 SDS) had severe *H19* DMR hypomethylation: mean *H19* DMR methylation was 2%, compared to 95% confidence interval 33–49.8% methylation in 95 healthy controls, and moderately reduced IGF2 DMR0 methylation: 27.6% compared to 36.5-54.9% in controls (Table 1 and Figure 1B). Subject 1 also had the highest fasting glucose which was in the impaired fasting glucose range, despite slim BMI (19.2 kg/m^2^) and young adult age (20 years).

All four subjects had a normal karyotype and no mutations in the *IGF2* gene were detected.

## Discussion

We describe the first case of severe hypomethylation of the *IGF2H19* DMR associated with severe pre-natal and post- natal growth restriction, insulin resistance, polycystic ovarian syndrome and precocious pubarche without convincing features of SRS. The majority of studies to date suggest *H19* DMR hypomethylation is a specific cause of SRS because it has not been found in any non-syndromic SGA patients screened [3,11,13,27,28]. Many also report that the degree of hypomethylation is linked with severity of the phenotype [9,28-30]. However, there have been a few case reports of hypomethylation of H19 DMR associated with either hemi-hypertrophy alone [29,30] or milder intrauterine growth restriction and post natal growth restriction associated with a prominent forehead and triangular facies as the only clinical signs [31]. Ours is the first case reported without asymmetry or abnormal face shape suggestive of SRS, however, she did have less specific features of retrognathia and a squeaky voice. Nonetheless, SRS is a disorder with marked clinical heterogeneity, and many of the clinical features regress with age.

We chose to study three molecular mechanisms (chromosomal, methylation and sequence abnormalities) by which *IGF2* expression could have been reduced in 4 women selected to have a similar phenotype to our published case report [18] who had severe intrauterine growth restriction, precocious pubarche, short stature and insulin resistance, and was found to have a balanced translocation affecting her *IGF2* gene regulation. We found 1 case with IGF2/H19 hypomethylation to a similar extent as observed in cases with SRS. The association of premature pubarche and insulin resistance in SRS has not been evaluated to date. Based on our observation from this study, we hypothesise that in addition to affecting pre- and post- natal growth, *IGF2* in humans may have a causal role in timing of pubarche and insulin resistance. This remains to be evaluated in future studies involving patients with *IGF2/H19* hypomethylation.

The limitations of our analyses include the low sensitivity of routine karytoping in detecting small chromosomal rearrangements, and only exonic sequencing of the *IGF2* gene. A full G-banded karyotype is able to detect structural abnormalities (deletions, translocations, inversions and insertions) at a resolution of approximately 10 million DNA base-pairs. Screening for mutations in the *IGF2* gene including the intron-exon boundaries of all nine exons has not shown any abnormalities to date [32], hence is unlikely to be an important cause of *IGF2* inactivation in humans.

## Conclusions

Our case of growth restriction, premature pubarche and insulin resistance in the absence of body asymmetry or other features of SRS adds to the expanding phenotype of *IGF2/H19* methylation abnormalities. Further studies are needed to confirm whether growth restriction in association with premature pubarche and insulin resistance is a specific manifestation of loss of *IGF2* expression through *IGF2/H19* loss of methylation.

## Abbreviations

BWS, Beckwith Wiedemann syndrome, CpG, cytosine guanine base pair; DMR, Differentially methylated region; DNA, Deoxyribonucleic acid; IGF2, Insulin like growth factor-2; SRS, Silver Russell syndrome; PCR, Polymerase chain reaction; SRS, Silver Russell syndrome.

## Competing interests

The authors declare that they have no competing interests.

## Authors’ contributions

RM conceived of the study design and drafted the manuscript; LI recruited the study cases, helped with the data analysis and interpretation; ATH participated in the study design and provided the control patient samples; JT performed the pyrosequencing analyses and helped in data analysis and interpretation. All authors read and approved the final manuscript.

## Pre-publication history

The pre-publication history for this paper can be accessed here:

http://www.biomedcentral.com/1471-2350/13/42/prepub
